# Methimazole Associated Neutropenia in a Preterm Neonate Treated for Hyperthyroidism

**DOI:** 10.1155/2015/680191

**Published:** 2015-02-24

**Authors:** Dimitrios Angelis, Rita Ann Kubicky, Alan B. Zubrow

**Affiliations:** ^1^St. Christopher's Hospital for Children, Drexel University College of Medicine, Philadelphia, PA 19134, USA; ^2^Division of Neonatology, Department of Pediatrics, Texas Tech University Health Sciences Center, Odessa, TX 79763, USA

## Abstract

Maternal Graves' disease is relatively uncommon with an estimated incidence of 0.4%–1% of all pregnancies, but only 1–5% of newborns delivered to mothers with Graves' disease develop overt clinical signs and symptoms of hyperthyroidism. Here, we describe a case of a 1380-gram female neonate who was born at 30-week gestation to a mother with Graves' disease. Our patient presented with hyperthyroidism followed by transient hypothyroidism requiring treatment with levothyroxine. While hyperthyroid, she was treated with methimazole, iodine, and a beta-blocker. 20 days after the initiation of methimazole, she developed neutropenia. The neutrophil counts started to improve immediately after the initiation of the weaning of methimazole. To the best of our knowledge, this is the first case reported in the literature of methimazole induced neutropenia in a preterm infant being treated for neonatal Graves' disease.

## 1. Introduction

We describe a case of methimazole related neutropenia in a preterm neonate with hyperthyroidism, born to a mother with Graves' disease. The neutropenia was noted 20 days after the initiation of methimazole and improved within 10 days after decreasing the dose of the medication. To the best of our knowledge, this is the first case reported in the literature of methimazole induced neutropenia in a preterm infant being treated for neonatal Graves' disease.

## 2. Case Report

A 1380-gram female neonate was born at 30-week gestation to a G1P0 mother with hyperthyroidism from Graves' disease; she was first diagnosed one year earlier and was started on methimazole 4 months before conception. Routine prenatal maternal labs were unremarkable. The mother developed severe preeclampsia, complicated by pulmonary edema; thus, an emergency cesarean section was performed. The mother received prenatal steroids 4 hours prior to delivery.

At delivery, the baby appeared active without overt signs of hyperthyroidism, but she had evidence of respiratory distress. Her weight was 1380 grams (60%), her length was 39 cm (59%), and her head circumference was 28 cm (77%). Her initial chest radiograph was consistent with RDS; she was intubated and received 1 dose of surfactant and then she was extubated and placed on nCPAP. Apgar scores were 7 and 9 (at 1 and 5 minutes, resp.). She remained on CPAP for 1 day and then was transitioned to room air.

On DOL 2, she had several episodes of apnea and caffeine citrate was started. On DOL 3, she developed sinus tachycardia (HR 220 bpm). She was evaluated for hyperthyroidism. Thyroid function tests showed a high FT4 of 5.2 ng/dL (normal: 0.75–1.54 ng/dL) and a suppressed TSH of <0.05 mIU/L (0.8–8.2 mIU/L). She was started on atenolol 1 mg/kg/day once daily and iodine (as Lugol's solution, 126.5 mg/5 mL), 1 drop (8 mg) every 8 hours, via a nasogastric tube.

On DOL 9, the infant was transferred to St. Christopher's Hospital for Children. Upon admission the vital signs were remarkable only for tachycardia (HR 180–210 bpm). The physical examination was normal. An echocardiogram demonstrated a small VSD but normal function. A HUS was normal.

**Figure 1:**
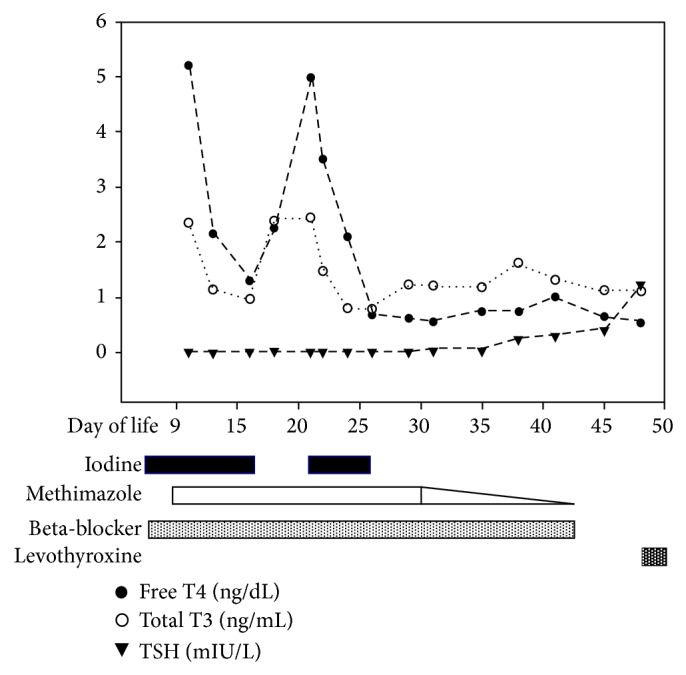
Free thyroxine (FT4) and thyroid stimulating hormone (TSH) levels and treatment course during hospitalization at our institution. Iodine, methimazole, beta-blocker, and levothyroxine are shown as bars at the end of the graph. The triangle at the end of methimazole bar represents the time that methimazole was started to be tapered.

On DOL 10, methimazole was started at 1 mg/kg/day in 3 divided doses, atenolol was discontinued, and propranolol was started at 1.5 mg/kg/day, in 3 divided doses, via a nasogastric tube. TSI and TBII were positive. During the next 5 days, thyroid hormone levels improved and the iodine solution was discontinued. A few days later, there was a rebound in the FT4 level, so the iodine was resumed and was able to be once again discontinued on DOL 25 (Figure 1).

**Figure 2:**
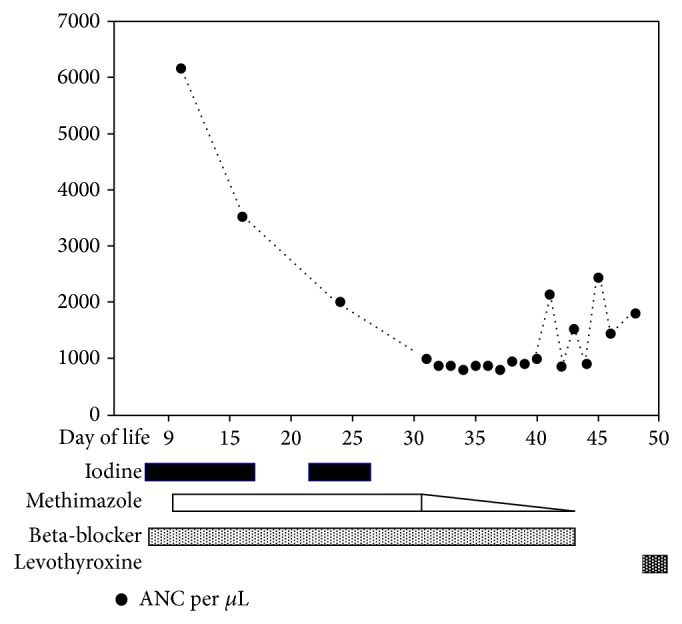
The absolute neutrophil count (ANC) is shown. Neutropenia was noted after 20 days of methimazole use (DOL 30). Iodine, methimazole, beta-blocker, and levothyroxine are shown as bars at the end of the graph. The triangle at the end of methimazole bar represents the time it was tapered.

Twenty days after the initiation of methimazole (DOL 30), she was noted to be neutropenic with an ANC of 990 cells/*μ*L, reaching a nadir of 795 cells/*μ*L between DOL 33 and 39 (Figure 2). Methimazole was gradually tapered starting from day 30 and stopped on DOL 44. The dose of methimazole was decreased by 20% every 2-3 days during which there was frequent monitoring of the TFT and white blood cell counts. By DOL 40, the ANC started to recover. When the daily dose of methimazole reached a third of the initial dose, it was maintained for five days and then discontinued (see Table 1).

She was discharged home on DOL 49 (37-week corrected age), weighing 2310 grams. At discharge the infant had an ANC of 1793 cells/*μ*L. The TFT was significant for a low FT4 level of 0.54 ng/dL but a normal TSH of 1.23 mIU/L. Due to her hypothyroidism, she was started on levothyroxine on a dose of 25 mcg PO daily. After discharge, on DOL 65, upon normalization of her TFT, levothyroxine was discontinued. Two weeks after the discontinuation of levothyroxine, repeat thyroid hormone levels were within normal limits. At her 2-year follow-up by her pediatrician, the patient had normal growth, premature thelarche, a language barrier, and developmental delay based on Denver Developmental Screening. She was receiving early intervention for speech and physical therapy.

## 3. Discussion

This is a case of methimazole induced neutropenia in a preterm neonate with neonatal hyperthyroidism, secondary to maternal Graves' disease. Maternal Graves' disease is relatively uncommon with an estimated incidence of 0.4%–1% of all pregnancies, but only 1–5% of newborns delivered to mothers with Graves' disease develop overt clinical signs and symptoms of hyperthyroidism [1, 2].

Methimazole and propylthiouracil (PTU) have been used as initial treatment of pediatric and neonatal Graves' disease. The mechanism of action of both drugs is to inhibit thyroid hormone synthesis by blocking thyroid peroxidase. This enzyme plays an important role in the iodination of the tyrosine residues in the thyroglobulin molecule and in the ultimate production of thyroxine and triiodothyronine. Both of these medications appear to be equally effective. Methimazole, though, is increasingly being used in the United States for the treatment of hyperthyroidism in children, because of the lower risk of hepatotoxicity compared to PTU [3]. The Federal Drug Administration has placed a black box warning against using PTU as a first line drug. PTU is still often used during the first trimester of pregnancy due to methimazole associated birth defects [1, 4].

Neutropenia is a serious, but rare, side effect of both methimazole and PTU. In adults, the estimated incidence of severe drug induced neutropenia is about 0.35% [5]. In the adult literature, most authors use the term agranulocytosis for severe cases of neutropenia when the absolute neutrophil count (ANC) is less than 500 cells/*μ*L, accompanied by fever, sore throat, and signs of sepsis. The definition of antithyroid drug induced neutropenia is not established in neonates and, therefore, the incidence is unknown. Graves' disease might be a cause of a mild neutropenia that is usually self-limited [6]. In our case the neutrophil count reached a nadir level of 795 cells/*μ*L.

Proposed mechanisms of methimazole induced neutropenia include production of autoimmune antibodies, hapten mediated autoimmunity, and a toxic effect on the bone marrow. Autoimmunity is thought to explain most cases of antithyroid drug induced neutropenia. This includes the presence of antineutrophil cytoplasmic antibodies (ANCA), a family of antibodies that are directed against neutrophil surface targets or cytoplasmic molecules (e.g., proteinase-3) [7]. Irrespective of the proposed underlying pathophysiology described in the adult literature, the toxicity of the antithyroid drugs appears to be related to the total dose that is administered to the patient. Higher and prolonged dosing schedules appear to increase the risk of neutropenia. Takata et al. [8] showed that adults with Graves' disease who are treated with 30 mg of methimazole daily developed agranulocytosis more frequently than those treated with 15 mg daily. In adults who received methimazole, but not PTU, Cooper et al. [9] showed that this effect was dependent on the dose and also on the age of the patient. Specifically, patients older than 40 years old were more likely to develop neutropenia. Similar trends and relations have not been described in neonates treated with methimazole.

The timing of the neutropenia is variable. Data from adults show that usually this occurs within 90 days from the initiation of methimazole [6, 10, 11]. Neutropenia might occur as early as 6 days [12] or as late as 10 years after the initiation of the drug [13]. In our case, neutropenia appeared early in the course of methimazole (20 days after the initiation of treatment) and the neutrophil counts started to improve immediately after the initiation of the weaning of methimazole. This suggests that neutropenia may be dose related and it makes the theory of direct bone marrow toxicity unlikely for this patient.

The recovery time of the neutrophil counts after the discontinuation of methimazole is approximately 10 days [10]. Cooper [6] suggests that the antithyroid medications should be stopped if the granulocyte count is less than 1000/*μ*L, with close monitoring in those patients with counts more than 1000 cells/*μ*L, but less than 1500/*μ*L. Although we did not stop the methimazole, despite the fall of the ANC to less than 1000 cells/*μ*L, we did follow a weaning protocol. We noted that the neutrophil count started to recover only by decreasing the dose of methimazole (by 20% every 2-3 days) and normalized by day of life 40. Our patient remained asymptomatic after day of life 25. Our observation that the neutrophil counts started to recover only when the dose of methimazole was decreased might be clinically relevant in neonates who develop neutropenia but remain hyperthyroid, despite maximal pharmacologic treatment. Reassuring in neonates who develop hyperthyroidism secondary to maternal Graves' disease is the knowledge that transplacentally acquired antibodies have a finite life span.

The management of neutropenia related to antithyroid medications in neonates is not established. Data from adults who have been treated with methimazole and who developed agranulocytosis show that stopping the medication is necessary for recovery [6]. The use of granulocyte colony-stimulating factor (G-CSF) has been shown to decrease the duration of neutropenia and achieve faster recovery [14]. This effect seems to be attenuated in cases of severe agranulocytosis with suppression of the bone marrow [15].

Pregnancy induced hypertension and preeclampsia are common complications of maternal hyperthyroidism. Our patient was delivered at 31-week GA, due to severe maternal preeclampsia. Millar et al. [16] showed that mothers with uncontrolled hyperthyroidism are at increased risk of severe preeclampsia. In cases though of uncontrolled hyperthyroidism there is also increased risk of prematurity, intrauterine growth restriction, and placental abruption. Mass screening (during or after pregnancy) may be important to prevent the deleterious effects of uncontrolled hyperthyroidism. It has been suggested that screening should be focused specifically on infants born to mothers with a previously affected infant, infants born to mothers who have received ablative treatment with radioiodine, or infants born to mothers with elevated maternal thyrotropin receptor antibodies at delivery [17]. The level of thyrotropin receptor antibodies at delivery (e.g., from cord blood) can predict the possibility of developing symptomatic hyperthyroidism in a newborn and could be used as a screening tool [18].

Babies born to mothers who were hyperthyroid during embryogenesis are at risk of congenital anomalies [19]. The presence of high levels of thyroid hormones affects the developing neonatal brain via various mechanisms that involve alterations in the production of neurotransmitter synthesis and the release of inflammatory cytokines [20]. In an animal model of induced hyperthyroidism, treatment with T3 resulted in decrease of growth of the cerebrum and to lesser degree of the cerebellum. The reduced brain size was caused by a permanent reduction in the final brain cell number [21]. There are limited data on long term developmental outcome of infants exposed to high levels of thyroid hormones in fetal or neonatal period. Hollingsworth and Mabry [22] reported four cases of congenital Graves' disease who had an autosomal dominant history of thyrotoxicosis and a possible gene mutation in the TSH receptor. Three out of four patients had growth delays and all had developmental delays upon follow-up. Our patient had a normal growth pattern at 2 years of age with no evidence of craniosynostosis, but she had a language delay which could be attributed to her exposure to abnormal levels of maternal thyroid hormones but also to her prematurity or growing up in a bilingual environment. Daneman and Howard [23] found varying degrees of intellectual impairment in six out of eight patients with neonatal hyperthyroidism with intellectual quotients ranging from 75 to 85. The six affected patients had craniosynostosis and all eight of them had normal physical growth.

Our patient presented with hyperthyroidism followed by transient hypothyroidism requiring treatment with levothyroxine. In a cohort of one preterm and three term neonates with overt hyperthyroidism, born to mothers with Graves' disease, Yael et al. [24] observed that FT4 peaked within the first 5 days of life, while TSH remained suppressed for up to three months. Central (pituitary) hypothyroidism following hyperthyroidism has been previously described in a preterm infant born to a mother with Graves' disease [25]. Kempers et al. [26] reported that all women with Graves' disease who gave birth to children with central congenital hypothyroidism were inadequately treated and that the incidence of central congenital hypothyroidism (due to prolonged suppression of the axis) secondary to maternal gestational hyperthyroidism was about 1 : 35,000. Since both hypothyroidism and hyperthyroidism are associated with adverse neurodevelopmental outcomes, more retrospective studies are needed to further evaluate and verify the neurocognitive development of patients with neonatal Graves' disease.

## Figures and Tables

**Table 1:** Free T4, total T3, and TSH levels as well as white blood cell count and absolute neutrophil counts during hospitalization at our institution. Normal values for FT4: 0.75–1.54 (ng/dL), T3: 0.9–2.6 (ng/mL), and TSH: 0.5–6.5 mIU/L.

Day of life	Free T4	Total T3	TSH	WBCs	ANCs
	(ng/dL)	(ng/mL)	(mIU/L)	(Cells/*μ*L)	(Cells/*μ*L)
10	5.2	2.36	0.02	14000	6160
12	2.16	1.15	0.01		
15	1.3	0.98	0.02	16700	3507
17	2.26	2.39	0.03		
20	4.99	2.45	0.02		
21	3.5	1.48	0.02		
23	2.1	0.8	0.02	18200	2002
25	0.7	0.79	0.02		
28	0.62	1.23	0.02		
30	0.57	1.21	0.04	16005	990
31				15200	870
32				17900	860
33				15900	795
34	0.75	1.19	0.05	13000	870
35				15000	860
36				20000	795
37	0.75	1.62	0.24	15700	942
38				15900	900
39				16500	990
40	1.01	1.32	0.31	19400	2134
41				17100	855
42				16800	1512
43				12800	896
44	0.66	1.13	0.42	14400	2440
45				12100	1444
47	0.54	1.12	1.23	16300	1793

## Abbreviations

ANC: Absolute neutrophil count
ANCA: Antineutrophil cytoplasmic antibodies
BPM: Beats per minute
DOL: Day of life
FT4: Free thyroxine
FDA: Federal Drug Administration
HR: Heart rate
HUS: Head ultrasound
nCPAP: Nasal continuous positive airway pressure
PTU: Propylthiouracil
RDS: Respiratory distress syndrome
T3: Total triiodothyronine
TBII: Thyrotropin-binding inhibitory immunoglobulin
TSH: Thyroid stimulating hormone
TSI: Thyroid stimulating immunoglobulin
TFT: Thyroid function test
VSD: Ventricular septal defect.
